# Collection, analysis and cryopreservation of semen from Malayan gaur (*Bos gaurus hubbacki*): A preliminary study

**Published:** 2012-10-31

**Authors:** M.I. Iswadi, Z.F. Ann, M.M. Hafiz, M.D. Hafiz, F.J. Fahrul, H. Hajarian, H. Wahid, I. Zawawi, M.S. Khairiah, O.A. Mazni

**Affiliations:** 1 *Veterinary Research Institute (VRI), Department of Veterinary Services, 59, Jalan Sultan Azlan Shah, 31400 Ipoh, Perak, Malaysia*; 2 *Agro-Biotechnology Institute, Malaysia (ABI), Ministry of Science, Technology and Innovation, P.O Box 341, Universiti Putra Malaysia, 43400 Serdang, Selangor, Malaysia*; 3 *Veterinary Hospital, Faculty of Veterinary Medicine, Universiti Putra Malaysia, 43400 Serdang, Selangor, Malaysia*; 4 *National Institute of Veterinary Biodiversity, Department of Veterinary Services, Bukit Dinding, 27000 Jerantut, Pahang, Malaysia*; 5 *Department Of Wildlife and National Parks, Pahang, Bangunan Asia Life, Jalan Teluk Sisek, 25000 Kuantan, Pahang, Malaysia*; 6 *Malaysian Agricultural Research and Development Institute (MARDI), 43400 Serdang, Selangor, Malaysia*

**Keywords:** Cryopreservation, Malayan gaur, Semen collection

## Abstract

The Malayan gaur (*Bos gaurus hubbacki*) or Seladang is classified as vulnerable by the International Union for Conservation of Nature and Natural Resources (IUCN). The Malayan gaur is mainly distributed in the tropical woodlands of Peninsular Malaysia and Southern Thailand. The aim of this study was to collect, analyze and cryopreserve the semen of wild Malayan gaur. Transrectal massage (TM) and electroejaculation (EEJ) technique was applied in semen collection of the Malayan gaur. The semen was then cryopreserved in liquid nitrogen using slow freezing technique. Makler counting chamber was used to evaluate sperm concentration and motility, while the sperm viability and morphology of fresh and post-thaw sperm was determined using eosin-nigrosin staining protocol. As a result, we have successfully collected the Malayan gaur semen using EEJ technique. Sperm motility, viability and morphological changes of the post-thaw semen of Malayan gaur were found undesirable due to the complication of the cryopreservation process. On the basis of current study it can be concluded that Malayan gaur bulls semen can be obtain by EEJ with no evidence of rectal trauma. Optimization of the process of cryopreservation for Malayan gaur sperm is needed to maintain the cryoviability of the good sperm quality. The data generated in this study would be useful in conservation of genetic diversity program for Malayan gaur.

## Introduction

The Malayan gaur (*Bos gaurus hubbacki*) or Seladang is mainly distributed in the tropical woodlands of Peninsular Malaysia and Southern Thailand (Lydekker, 1907). The gaurs are listed as vulnerable by the International Union for Conservation of Nature and Natural Resources (IUCN) (Duckworth *et al.*, 2008). Their usual diet comprises of grasses, shoots and fruits. The average life span of the gaur is about 30 years. Generally, the gaur is characterized by their body length (250-330 cm), shoulder height (170-220 cm), tail length (70-100 cm) and average weight (1000-1300 kg) (IUCN, 2010). Their whole body is covered with a dark brown coat, while their lower legs vary from white to tan in colour.

In both sexes, the horns grow from the sides of their head and curve upwards. A bulging grey-tan ridge can be seen on the forehead connecting the horns and the forehead. Shoulder humps are present in adult males but none in adult females. The gestation period of the gaur is about 310 to 314 days. Normally, an adult female gaur gives birth to one calf per pregnancy (Hubback, 1937). Naturally, the gaur is diurnal, being most active in the morning and late afternoon, resting during the midday. To date, the average population density of the gaur is about 0.6 animals per square kilometer with herds having home ranges of around 80 kilometers square. The gaur lives in herds led by a single adult male with small mixed herds of 2 to 40 individuals (IUCN, 2010).

In Malaysia, the population of the gaur was stated to be less than 500 individuals and it is thought to be declining (Read *et al.*, 1994). In Taman Negara, the density of the gaurs were crudely estimated from surveys between 1999 to 2001 as 0, 3 and 22 gaurs per 100 km² (Kawanishi and Sunquist, 2004). Furthermore, the mortality rate is quite high in 1998 and 1999 at 18% and 23% respectively (Sahir, 2001).

In terms of semen storage of threatened species, considerable attention has been given to the determination of optimum conditions for collection and freezing of the semen of Malayan gaur. Previously, various techniques were applied in semen collection of domestic and wild species under threat (eg; artificial vagina, transrectal massage (TM), electroejaculation (EEJ) and etc.) (Schmitt and Hildebrandt, 1998). TM and EEJ were mostly considered to be the suitable option to collect sperm from the untrained wild species as compared to domestic bulls (Palmer *et al*, 2005).

Semen cryopreservation offers many advantages to the maintaining sperm storage of the threatened wild species which provide genetics resource bank in animal conservation (Fickel *et al*, 2007). Cryopreservation is a technique that involves a very wide range of temperature changes. However, the biggest obstacle to exploiting cryopreserved semen of many species is that cooling, freezing and thawing which generally damage sperm membrane structures, causes fewer viable and motile sperm (Hammerstedt *et al*, 1990). Despite reducing the sperm motility, cryopreservation processes was found to be the factor induced morphological changes of the sperm including damage to mitochondria and sperm tail which cause impairment to fully sperm function (O’Connell *et al*, 2002).

Due to the vulnerable existence of the Malayan gaur, this study was designed with an objective to collect, analyze and cryopreserve the Malayan gaur semen for the conservation program.

## Materials and Methods

### Semen collection

Fresh semen samples were collected from three male adult Malayan gaur at Jenderak Selatan Wildlife Conservation Centre, Department of Wildlife and National Parks, Peninsular Malaysia by TM and EEJ technique. After three weeks of the first semen collection, the semen samples were collected again using the same semen collection technique.

The TM technique involved the operator inserting a hand into the rectum and then alternately massaging the ampullae firmly followed by rhythmically stroking the urethralis muscles. The semen was collected as it was emitted from the preputial orifice into a graduated test tube.

The EEJ technique was accomplished using an automated semen collection unit with automatic and manual settings (ElectroJac5, Ideal Instruments, Neogen Corporation, USA) and a 66-mm rectal probe with three ventrally oriented electrodes. Immediately after collection, the progressive motility was evaluated under a light microscope at 100x magnification. The volume of each sample was measured in a graduated test tube. A Makler counting chamber (Sefi-Medical Instruments Ltd) was used to determine the sperm concentration. Subsequently Bioxcell® medium (IMV Technologies, France) was gently added to all samples before proceeding the cryopresevation process. Bioxcell® medium provides nutrients and contains cryoprotectant agent (CPA) to extend the longevity of the sperm during the cryopreservation process. Progressive motility, viability and morphology of the sperm were later determined in fresh and post-thaw sample.

### Cryopreservation technique

In this study, slow freezing technique or 2-step freezing technique was used. After adding Bioxcell® medium, the samples were then put in a 4°C cooling cabinet for 4 hours. Then the samples were aspirated using a 0.25-ml straw with a vacuum pump attached with filling nozzle. After 9 minutes above the liquid nitrogen vapor (-60°C to -120°C), the straws were then plunged and stored in liquid nitrogen (-196°C). After 24 hours, the semen was thawed at 37°C for 60 seconds. The semen was then examined for motility, viability and morphology of the sperm.

### Motility assessment

A Makler counting chamber (Sefi-Medical Instruments Ltd) was used to determine the sperm motility. Sperm motility was graded from a to d, according to the World Health Organization (WHO, 1999) Manual criteria as follows (a for fast progressive motility; b for slow progressive motility; c for non-progressive sperm; d for immotile sperm).

### Viability and morphology evaluation

Sperm viability and morphology were determined using eosin–nigrosin staining protocol with bright field microscopy at 400x magnification. Normal sperm morphology should have a regular oval head with a connecting midpiece and long straight tail. Kruger’s strict criteria is used to evaluate sperm morphology (≥15% normal: normal range - good prognosis; 5-14% normal: sub-optimal range - prognosis is fair to good; 0-4% normal: poor prognosis). A normal sample should have at least 15% normal forms. Whereas, in viability assessment sperm that were white or unstained were classified as live, while those that showed red coloration in the head region were considered dead with bright field microscopy at 400x magnification. Result for viability test is recorded in live : dead ratio. At least 200 sperm were assessed for each preparation.

## Results

### Transrectal massage vs electroejaculation

Only one out of the three Malayan gaur bull had any evidence of semen during an attempt to extract by TM. However, that sample showed high levels of urine contamination. The contaminated semen was discarded. Following EEJ technique by electrical stimulation, the semen was collected from all three Malayan gaur. There was no evidence of rectal trauma by electrical stimulation in all those three Malayan gaur. All Malayan gaur in this study achieved an erection.

During electrical stimulation, musculoskeletal movement consisted of hindlimb extension and pelvic thrusting which is typical when using EEJ. By using the EEJ, the first Malayan gaur ejaculated a very large volume of semen without gross urine contamination with high progressively motile sperm. The second Malayan gaur ejaculated a large volume of semen without gross urine contamination and very minimal debris with high progressively motile sperm. However, the third Malayan gaur ejaculated only a small volume of semen but without gross urine contamination and with high progressively motile sperm.

### Evaluation of semen quality

Briefly, semen volume ranged 0.3 ml to 11.0 ml (mean 5.65 ml) and 0.2 ml to 8.5 ml (mean 4.35 ml) when collected by TM and EEJ respectively. pH level in collected semen by TM range from 8.22 to 8.42 and EEJ range from 6.58 to 7.95.

However, no progressive motile sperm has been seen in semen collected following TM (0%) when compared to the EEJ technique (16%-70%) (Table 1).

**Table 1:** Percentage motility of fresh and post-thaw bull semen collected by EEJ and TM in first and fourth week.

Weeks	Semen collection technique	Bull No.	No. of semen collections	Motile (%)	
				fresh semen	post-thaw semen
1	EEJ	1	1	0	0
1	EEJ	1	2	0	0
1	EEJ	1	3	0	0
1	EEJ	1	4	0	0
1	EEJ	1	5	60	30
1	EEJ	1	6	70	20
1	EEJ	2	1	16	10
1	EEJ	3	1	0	0
1	EEJ	3	2	0	0
4	TM	2	1	0	0
4	TM	2	2	0	0
4	TM	2	3	0	0
4	EEJ	2	1	0	0
4	EEJ	2	2	70	35
4	EEJ	3	1	70	40

### Effect of cryopreservation on motility, viability and morphology

**Fig. 1:**
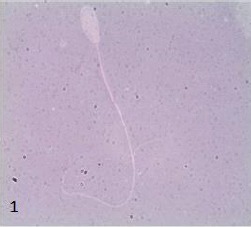
Patterns of live and viable Malayan gaur sperm stained with eosin-nigrosin at 1000x magnification. Viable sperm can be identified as having a clear white or unstained head region.

**Fig. 2:**
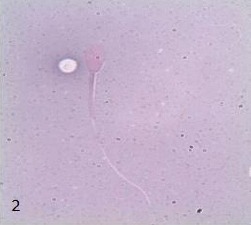
Patterns of dead and non-viable Malayan gaur sperm stained with eosin-nigrosin at 1000x magnification. Non-viable sperm shows red coloration in the head region.

The post-thawed sperm motility decrease in a range from 10% to 40% as compared to sperm motility in fresh semen (16% to 70%). Whereas, in viability assessment, sperm that were white or unstained were classified as live, while those that showed red coloration in the head region were dead (Figures 1 and 2).

There were more viable sperm in fresh semen (86%) as compared to the post-thaw semen (64%) (Table 2). About 87.5% of normal sperm morphology was seen in fresh semen compared to post-thaw semen (87%) (Table 3).

**Table 2:** Bull sperm viability in fresh and freeze-thawed samples.

	Fresh	Post-thaw
Live (n)	85.92% (n=177)	63.77% (n=132)
Dead (n)	14.08% (n=29)	36.23% (n=75)
Live : Dead Ratio	6.1 : 1.0	1.8 : 1.0
Viability (%)	86	64
Total sperm (n)	206	207

**Table 3:** Individual bull sperm morphology in fresh and freeze-thawed samples.

	Fresh		Post-thaw	
	
	n	%	n	%
Normal	175	87.5	174	87.0
Coiled tail	8	4.0	8	4.0
Detached head	9	4.5	3	1.5
Proximal droplet	1	0.5	6	3.0
Bent tail	11	5.5	8	4.0
Big neck	0	0.0	1	0.5
Coiled midpiece	2	1.0	7	3.5
Total	200	100	200	100

Normal sperm morphology should have a regular oval head with a connecting midpiece and long straight tail whilst abnormal sperm was identified having coiled tail, detached head, proximal droplet, bent tail, big neck and coiled midpiece.

## Discussion

According to Palmer *et al.*, (2004), TM directed specifically towards the ampullary region of the ductus deferens and has shown to be very effective for producing semen emission and semen samples. Similarly, Parsonson *et al*. (1971) have been reported a TM method also used for collection of seminal fluids for microbiologic examination and suggested that the TM method could also be used for semen evaluation in bulls. The TM method also has been reported to be used for semen collection in cattle (McGowan *et al.*, 1995; Wolfe, 2001), elephants (Schmitt and Hildebrandt, 1998) and man (Fahmy *et al.*, 1999). Usually the TM method can be an optional artificial vagina for semen collection on untrained bulls. Hence, semen collection by the TM method might be a suitable method to be used under field conditions by veterinary practitioners whereas other semen collection methods such as artificial vagina is are less suitable.

However, the results in this study were shown that semen collection in untrained Malayan gaur by TM method is difficult to be applied due to stress on the animal. This was supported by Palmer *et al.*, (2004) who reported that handling stress is a major limiting factor for successful semen collection by TM. Furthermore, in our study unlike the EEJ technique, no semen was collected by the TM method. However, if the semen was successfully collected by TM, the semen will had lower sperm concentration, lower percent motile and live sperm than samples collected by EEJ (Palmer *et al.*, 2005). This is due to the prolonged exposure of sperm to air, temperature and environment in the distal preputial cavity which reflect to the low motility and viability in the semen samples collected by TM (Persson *et al.*, 2006).

When the EEJ technique is performed by a skilled veterinarian, it is most likely to result in a semen collection in more than 95% of the bulls (McGowan, 2004). The EEJ technique is routinely used in many countries since it is considered to be a quick, safe and reliable procedure. Therefore, EEJ technique could be an alternative for semen collection despite welfare considerations is needed due to stress or pain of EEJ in many countries (Falk *et al.*, 2001). Furthermore, EEJ was more likely to result in successful semen collection (100%) compared with TM (80%) which the semen was successfully collected by EEJ from all bulls in which TM had failed (Persson *et al.*, 2006). In this study, we show that EEJ technique can be used for semen collection of Malayan gaur instead of TM.

Immediately after the Malayan gaur semen has been collected, the progressive sperm motility and sperm concentration are assessed. In this study, the results show the percentage of progressive motile sperm is about 16% to 70% in range. The result in our study is comparable with the report by Barth (2000) where an acceptable semen sample collected in the field by EEJ was shown to have 40% to 59% individual motile spermatozoa (60% to 69% for a good sample and 80% to 100% for a very good sample). In sperm concentration evaluation, the acceptable sperm concentration should have exceeding 250 million sperm per mL (a good sample at least 400 million sperm per mL and a very good sample at least 750 million sperm per mL). As compared to this study, the sperm concentration is recorded between 400 to 10200 million sperm per mL which considered being a very good sample.

Based on our results, the pH of freshly ejaculated semen was high in semen by collected by TM (8.22 to 8.42) when compared to EEJ (6.58 to 7.95). The exact cause of abnormal level of semen pH in TM is unkown. Normally, semen secretion contain basic amines which have alkaline bases such as, putrescine, spermine, spermidine and cadaverine which are responsible for smell and flavor of the semen. These alkaline bases are needed to counteract the acidic environment of the vagina canal and protect the DNA inside the sperm from acidic denaturation. In contrast, Aghangari (1992) found that the decrease in the pH level was due to sperm cells actively producing more lactic acid. Bearing in mind, an abnormally high or low semen pH can affect the sperm ability to move or to penetrate an egg.

Recently Fatimah *et al*. (2010) were successful in cryopreserving sperm from domestic species using slow freezing cryopreservation protocol. However, cryopreservation protocols vary among species due to the differences in shape, volume, organelles size and composition of the sperm (Purdy, 2006). Thus, when a cryopreservation protocol has been optimized for sperm of one species, it may not be ideal for sperm of other species. Our study shows that the cryopreservation by slow freezing of the Malayan gaur sperm was found to reduce the motility and viability of sperm when compared to the sperm in fresh semen. This was supported by Salamon and Maxwell (2000) in their report where the main changes that occur during cryopreservation are mainly ultrastructural, biochemical and functional, which will impair sperm transport and survival. As a result, 50% of bull sperm viability has been established and accepted as the usual percentage of sperm damaged during cryopreservation (Rasul *et al.*, 2007).

On the other hand, cryopreservation is a well known cause of defective sperm by decreasing factors such as motility (Barbas and Mascarenhas, 2009) and membrane damage (Hammerstedt *et al.*, 1990). Progressive motility before freezing in this study ranged from 16% to 70% (mean 57.2%), which is somewhat higher than the results of Cary *et al*. (2004) and Braun *et al*. (1994), who obtained pre-freezing motility rates of 4% to 62% (mean of 33%) and 25% to 75% (mean 50%) following collection from normal stallion species. The progressive motility following thawing of cryopreserved ejaculated sperm was 10% to 40% (mean 27%), comparable with 1% to 43% (mean 16.3%), obtained by Cary *et al*. (2004).

As reported by Stradaioli *et al*. (2007), the use of Bioxcell® in domestic bulls has the ability to maintain glutathione content in cryopreserved sperm because it contains higher concentrations of glutathione than tris-egg yolk extender (450 µmoles vs 40 µmoles). Furthermore a positive relationship has been reported between glutathione levels and semen quality (Gadea *et al.*, 2004), and it is suggested in improvement of post-thaw quality of bovine semen (Stradaioli *et al.*, 2007). Cryopreserved of bovine semen in Bioxcell® may be attributed to the higher concentrations of glutathione which protect the sperm against oxidative stress and reactive oxygen species. In Malayan gaur semen, Bioxcell® could improve semen quality as seen in our results. It is suggested that Malayan gaur bull sperm is less prone to oxidative stress due to low contents of polyunsaturated phospholipids. Therefore, the level of glutathione in Bioxcell® is sufficient enough to protect the sperm from oxidative stress during freeze-thawing process.

The surviving sperm population might have morphological defects that reduce and impairs their fertilizing ability (Correa *et al.*, 2007). In our study, the sperm morphology was evaluated either directly in the field or at the laboratory. One of the most accepted definitions of normal sperm morphology is that at least 70% of the sperm should be normal (Fitzpatrick *et al.*, 2002). While, according to the WHO and Kruger’s strict criteria, the morphological normal sperm should not be less than 30% and 15% respectively (Kruger *et al.*, 1988; WHO, 1999). Abnormal morphological Malayan gaur sperm was identified as having coiled tail, detached head, proximal droplet, bent tail, big neck and coiled midpiece. Although there was a decrease of morphological normal sperm in cryopreserved semen compared to fresh semen, the percentage of morphological normal sperm is acceptable to define that there were high morphologically normal sperm in our samples according to WHO and Kruger’s strict criteria.

Semen quality was reported to be dependent on many factors, including seasonal changes, sexual activity and individual variability (Blottner *et al.*, 2001). Therefore, success in collection and cryopreservation of limited amounts of semen may be challenging. To our knowledge, there is very limited data about the collection and cryopreservation of Malayan gaur sperm and their relationship with the pregnancy rates. Further advances in optimizing the semen collection and cryopreservation of Malayan gaurs are required to improve the utilization and storage of collected sperm.

## Conclusion

On the basis of current study it was concluded that, i. Malayan gaur bulls semen can be obtain by EEJ with no evidence of rectal trauma. ii. Following EEJ technique, the semen quality (volume, pH, progressive motile) is better as compared to TM. iii. Bioxcell® was able to give a good results for motility, viability and morphology of sperm after freezing and thawing. Further study on collection of semen by TM or EEJ, analysis and cryopreservation of semen using various cryoprotectants for the genetic preservation of Malayan gaur will bring more useful information in the future.
